# Heart Rate Variability in Dental Science

**DOI:** 10.3389/fmed.2019.00013

**Published:** 2019-02-06

**Authors:** Robert L. Drury, Scott A. Simonetti

**Affiliations:** ^1^ReThink Health, Bainbridge Island, WA, United States; ^2^Institute for Discovery, University of Wisconsin, Madison, WI, United States; ^3^Advanced Facialdontics LLC, New York, NY, United States

**Keywords:** heart rate variability, research priorities, dental assessment, dental procedures, dental science

## Abstract

Dentistry has made progress as a profession by integration with both medicine and other human sciences, especially when it uses empirical metrics to study process and outcome variables. Notably, progress in our understanding of genomic, biomic, and other molecular biological phenomena has been valuable. As has been identified by Drury (1, 2), it is proposed in this commentary that the inclusion of heart rate variability (HRV) as a biomarker of health may further this integrative progress. HRV is derived by various linear and non-linear statistical analyses of the R-R, beat-to-beat ECG interval in microseconds. Over twenty three thousand reports are identified in a recent PubMed search of the term heart rate variability, most of which demonstrate HRV's sensitivity to a wide diversity of physical and psychosocial pathologies. The small literature of dental use of HRV in both assessment and treatment will be selectively reviewed and relevant exemplars for other important health applications of HRV will be discussed. This will lead to a proposed agenda for researching HRV's value to professional dentistry as a human health and wellness profession.

Before HRV was specifically identified, it was well-known that dentistry involved negative affective states such as fear, stress, and pain. Indeed, “painless dentistry” was a key marketing feature of some practitioners, and “gentle dentistry” is still a frequent practice name. Although originally using rather unsophisticated unidimensional approaches, our increasing knowledge of biomedicine, neuroscience, and psychosocial issues has led to recent findings summarized by Flaten and al Absi (3) on the cardiovascular and neuroendocrine elements of response to stressful dental treatments. Similarly, the effects of dental surgery on cardiovascular and sympathetic responses have been observed (4), as well as deep pressure input on the parasympathetic system in wisdom tooth extraction (5).

As awareness and understanding of HRV have matured, as in this Research Topic, it has been used in a variety of ways to aid in both assessment and treatment of dental issues and conditions (6). For example the suppression of HRV in malocclusion has been demonstrated by Ekuni et al. (7) and the non-linear characteristics of HRV during endodontic treatment have been identified (8). HRV has been used to monitor orthostatic dysfunction during postural change in the dental chair (9). Several studies have used HRV analysis in the treatment of burning mouth syndrome (10, 11).

In an especially salient study, the OPPERA (Orofacial Pain: Prospective Evaluation and Risk Assessment) case-controlled study followed 1633 Tempero Mandibular Dysfunction-free controls and 185 TMD cases for several years at multiple dental universities. The primary finding that emerged from the initial studies of potential risk factors was relative to controls, TMD cases displayed a dysfunction in autonomic activity characterized by reduced HRV at rest and in response to both physical and psychological stressors (12). This was reflected by a decrease in all HRV measures in both time and frequency domain during each of the test epochs. There is emerging evidence that somatosensory disorders such as TMD and Fibromyalgia are associated with a reduction in HRV and an overall dysfunction in autonomic activity (13). The association between TMD and headaches (14) coupled with the evidence that headache patients also have increased sympathetic nervous system activity and decreased parasympathetic activity compared to non-headache controls (15), clarifies the emerging need to measure and monitor HRV in dentistry. TMD, being one of the most common chief complaints a dentist may encounter, has always been thought of as a “stress” syndrome, but HRV now provides a quantitative method to assess, diagnose, and measure treatment effectiveness. Addressing, and possibly improving a biomarker such as HRV via a common dental disorder such a TMD, will allow for the potential improved understanding of comorbid symptoms such as migraine and tension headaches, myofascial pain, and fibromyalgia. With the decreasing cost and increasing accuracy of modern biosensors, it is realistic to expect HRV monitoring to become ubiquitous within the dental profession to enhance and measure treatment process and efficacy.

Beyond the use of HRV in assessment of dental processes and interventions, it has been used in applications which include treatment for physical disorders and psychosocial conditions. HRV Biofeedback (HRVB) has been studied in a number of clinical applications in cardiac rehabilitation and impaired sleep quality (16). HRVB has also shown value in modulating emotional response (17, 18) and treating disorders such as depression (19), stress (20), pain (21), post-traumatic stress disorder (22), and substance use disorder (23). Conversely, exercise therapy has been studied empirically and found to improve HRV (24) and some pharmacological agents have been shown to improve HRV (25, 26), as has hypnosis (27), message (28), and yoga (29). These results are encouraging but they have not been applied to dental treatment issues as of yet.

This general commentary has highlighted the rapidly emerging body of findings showing HRV to be a significant biomarker of various human health conditions, diseases and important functional states. Applications of such findings to dental practice have not been made in the dental literature and this should be a high priority. Not only is HRV of great potential use in assessing and evaluating various dental conditions and procedures, but it has also been helpful in treating a wide variety of non-dental conditions, and may well-generalize in dental treatment. In particular, rapid advances in wearable biosensors, networked algorithmic streamed data analysis and feedback, machine learning, artificial intelligence, and epigenetics will strongly contribute to the overall development of the fields of human health and well-being. This period of rapid technological development (2, 30) has been identified in the current Research Topic and presents a major opportunity for dental science.

## Author Contributions

RD conducted the overview of heart rate variability literature relevant to dental practice. He identified contributions and the need for additional research in this area. SS reviewed the manuscript.

### Conflict of Interest Statement

The authors declare that the research was conducted in the absence of any commercial or financial relationships that could be construed as a potential conflict of interest.
